# Supplementary material for: Effectiveness of first-line treatment for relapsing-remitting multiple sclerosis in Brazil: A 16-year non-concurrent cohort study
Source: PLoS One. 2020 Sep 2;15(9):e0238476. doi: 10.1371/journal.pone.0238476 (PMC7467258; doi:10.1371/journal.pone.0238476)
Supplement: S1 Table — (DOCX) [file pone.0238476.s001.docx]

Table S1. Characteristics of Brazilian patients with RRMS included in the study after matching by propensity score, 2000-2015.

|  | **βINF- 1a IM vs. βINF-1b SC**  **βINF-1b SC** | | **βINF-1a IM vs. GA**  **GA** | | **βINF- 1a IM vs. βINF-1a SC**  **βINF-1a SC** | | **βINF-1b SC vs. GA**  **GA** | |
| --- | --- | --- | --- | --- | --- | --- | --- | --- |
| **Gender** |  |  |  |  |  |  |  |  |
| Female, n (%) | 2,923 (71.5) | 2,813 (68.8) | 3,029 (76.8) | 3,011 (76.3) | 4,154 (74.3) | 3,940 (70.4) | 2,641 (71.5) | 2,791 (75.6) |
| Male, n (%) | 1,167 (28.5) | 1,277 (31.2) | 915 (23.2) | 933 (23.7) | 1,440 (25.7) | 1,654 (29.6) | 1.052 (28.5) | 902 (24.4) |
| **Age** |  |  |  |  |  |  |  |  |
| Mean (SD)  n (DP) | 37.7 (±11.4) | 37.8 (±11.6) | 37.1 (±11.3) | 37.2 (± 11.3) | 37.1 (± 11.2) | 38.0 (± 11.5) | 37.7 (± 11.4) | 37.4 (±11.5) |
| **Age Group, n (%)** |  |  |  |  |  |  |  |  |
| 18-25 | 595 (14.5) | 627 (15.3) | 620 (15.7) | 619 (15.7) | 878 (15.7) | 848 (15.2) | 568 (15.4) | 599 (16.2) |
| 26-35 | 1,298 (31.7) | 1,268 (31.0) | 1,284 (32.5) | 1,330 (33.7) | 1,854 (33.1) | 1.714 (30.6) | 1,153 (31.2) | 1,227 (33.2) |
| 36-45 | 1,165 (28.5) | 1,101 (26.9) | 1,121 (28.4) | 1,044 (26.4) | 1,559 (27.9) | 1,501 (26.8) | 1,014 (27.4) | 948 (25.7) |
| 46-55 | 730 (17.8) | 795 (19.4) | 657 (16.7) | 676 (17.1) | 933 (16.7) | 1,114 (19.9) | 708 (19.2) | 644 (17.4) |
| 56-65 | 258 (6.3) | 254 (6.2) | 224 (5.7) | 242 (6.1) | 317 (5.7) | 346 (6.2) | 211 (5.7) | 242 (6.6) |
| > 65 | 44 (1.2) | 45 (1.2) | 38 (1.0) | 38 (1.0) | 53 (0.9) | 71 (1.3) | 39 (1.1) )) | 33 (0.9) |
|  |  |  |  |  |  |  |  |  |
| **Period of cohort entry, n (%)** |  |  |  |  |  |  |  |  |
| 2000-2003 | 428 (10.5) | 470 (11.5) | 306 (7.7) | 286 (7.3) | 428 (7.7) | 432 (7.7) | 293 (7.9) | 286 (7.5) |
| 2004-2007 | 1,061 (25.9) | 1,025 (25.1) | 957 (24.3) | 899 (22.8) | 1,358 (24.3) | 1,701 (30.4) | 949 (25.7) | 836 (22.6) |
| 2008-2011 | 1,799 (44.0) | 1,826 (44.6) | 1,596 (40.5) | 1,663 (42.2) | 2,167 (38.7) | 2,254 (40.3) | 1,694 (45.9) | 1,603 (43.4) |
| 2011-2015 | 802 (19.6) | 769 (18.8) | 1,085 (27.5) | 1,096 (27.7) | 1,641 (29.3) | 1,207 (21.6) | 757 (20.5) | 968 (26.2) |
|  |  |  |  |  |  |  |  |  |
| **Region of residence,**  **n (%)** |  |  |  |  |  |  |  |  |
| North | 49 (1.2) | 76 (1.8) | 27 (0.7) | 34 (0.9) | 52 (0.9) | 72 (1.3) | 358 (1.0) | 326 (0.9) |
| Northeast | 495 (12.1) | 569 (13.9) | 366 (9.2) | 404 (10.2) | 590 (10.6) | 875 (15.6) | 472 (12.8) | 404 (10.9) |
| Midwest | 378 (9.3) | 447 (10.9) | 300 (7.6) | 322 (8.2) | 418 (7.5) | 657 (11.7) | 358 (9.7) | 326 (8.8) |
| Southeast | 2,450 (59.9) | 2,296 (56.2) | 2,420 (61.4) | 2,345 (59.5) | 3,257 (58.2) | 3,107 (55.5) | 2,184 (59.1) | 2,229 (60.4) |
| South | 718 (17.5) | 702 (17.2) | 831 (21.1) | 839 (21.2) | 1,277 (22.8) | 883 (15.9) | 643 (17.4) | 700 (19.0) |
| **Elixhauser**  **Comorbidities, n (%)**  **n (%)** |  |  |  |  |  |  |  |  |
| 0 | 3,660 (89.5) | 3,581(87.6) | 3,522 (89.3) | 3,515 (89.1) | 5,060 (90.5) | 4,947 (88.5) | 3,254 (88.1) | 3,287 (89.0) |
| 1 | 391(9.6) | 471(11.5) | 383 (9.7) | 380 (9.6) | 486 (8.6) | 591 (10.5) | 404 (10.9) | 363 (9.8) |
| ≥2 | 39 (0.9) | 38 (0.9) | 39 (1.0) | 49 (1.3) | 480 (0.9) | 56 (1.0) | 35 (1.0) | 43 (1.2) |
| **Exclusive SUS patients, n (%)** | 1,806 (44.2) | 2,005 (49.0) | 1,508 (38.2) | 1,567 (39.7) | 2,005 (35.8) | 2,556 (45.7) | 1,682 (45.5) | 1,521 (41.2) |
|  |  |  |  |  |  |  |  |  |

|  | **βINF-1b SC vs. βINF-1a SC**  **βINF-1a SC** | | **GA vs. βINF-1a SC**  **βINF-1a SC** | |
| --- | --- | --- | --- | --- |
| **Gender** |  |  |  |  |
| Female. n (%) | 3,565 (70.3) | 3,449 (68.1) | 3,015 (76.4) | 2,588 (65.6) |
| Male. n (%) | 1,502 (29.7) | 1,618 (31.9) | 933 (23.6) | 1360 (34.4) |
| **Age** |  |  |  |  |
| Mean (SD)  n (DP) | 38.2 (±11.2) | 38.3 (± 12.0) | 37.3 (± 11.3) | 39.2 (± 11.7) |
| **Age Group, n (%)** |  |  |  |  |
| 18-25 | 710 (14.0) | 817 (16.1) | 619 (15.8) | 558 (14.2) |
| 26-35 | 1,512 (29.8) | 1,440 (28.4) | 1,332 (33.7) | 1,038 (26.3) |
| 36-45 | 1,483 (29.2) | 1,343 (26.5) | 1,046 (26.5) | 1,111 (28.1) |
| 46-55 | 1,026 (20.2) | 1,035 (20.4) | 676 (17.1) | 906 (22.9) |
| 56-65 | 285 (5.6) | 353 (6.9) | 242 (6.1) | 279 (7.1) |
| > 65 | 51 (1.2) | 79 (1.7) | 33 (0.8) | 56 (1.4) |
|  |  |  |  |  |
| **Period of cohort entry.**  **n (%)** |  |  |  |  |
| 2000-2003 | 1,420 (28.0) | 1,408 (27.8) | 286 (7.2) | 289 (7.3) |
| 2004-2007 | 1,031 (20.3) | 1,052 (20.8) | 899 (22.8) | 1,329 (33.7) |
| 2008-2011 | 1,847 (36.5) | 1,394 (27.5) | 1,667 (42.2) | 1,368 (34.7) |
| 2011-2015 | 769 (15.2) | 1,213 (23.9) | 1,096 (27.8) | 962 (24.3) |
|  |  |  |  |  |
| **Region of residence, n (%)** |  |  |  |  |
| North | 93 (1.8) | 131 (2.6) | 34 (0.9) | 43 (1.1) |
| Northeast | 613 (12.1) | 709 (14.0) | 404 (10.2) | 673 (17.1) |
| Midwest | 509 (10.1) | 619 (12.2) | 326 (8.2) | 455 (11.5) |
| Southeast | 2,987 (58.9) | 2,603 (51.4) | 2,345 (59.4) | 2,224 (56.3) |
| South | 865 (17.1) | 1,005 (19.8) | 839 (21.3) | 553 (14.0) |
|  |  |  |  |  |
|  |  |  |  |  |
| **Elixhauser comorbidities, n (%)** |  |  |  |  |
| 0 | 4,509 (89.0) | 4,495 (88.7) | 3,515 (89.0) | 3,526 (89.3) |
| 1 | 512 (10.1) | 520 (10.3) | 384 (9.7) | 386 (9.8) |
| ≥2 | 46 (0.9) | 52 (1.0) | 49 (1.3) | 36 (0.9) |
| **Exclusive SUS patients, n (%)** | 2,468 (48.7) | 2,385 (47.1) | 1,571 (39.8) | 2,106 (53.3) |
|  |  |  |  |  |

**Table S1** (continued)

**Abbreviations:** βIFN-1a IM**:** IM-IFNβ-1a. intramuscular interferon beta-1a; SC-IFNβ-1b. subcutaneous interferon beta-1b; SC-IFNβ-1a. subcutaneous interferon beta-1a; GA. Glatiramer acetate; SUS. Brazilian Public Health System
