# Supplementary material for: Effectiveness of first-line treatment for relapsing-remitting multiple sclerosis in Brazil: A 16-year non-concurrent cohort study
Source: PLoS One. 2020 Sep 2;15(9):e0238476. doi: 10.1371/journal.pone.0238476 (PMC7467258; doi:10.1371/journal.pone.0238476)
Supplement: Attachment — Submitted filename: Response to Reviewers.docx [file pone.0238476.s002.docx]

**Response to Reviewers**

Reviewers' comments:

Reviewer's Responses to Questions

Comments to the Author

1. Is the manuscript technically sound, and do the data support the conclusions?

The manuscript must describe a technically sound piece of scientific research with data that supports the conclusions. Experiments must have been conducted rigorously, with appropriate controls, replication, and sample sizes. The conclusions must be drawn appropriately based on the data presented.

Reviewer #1: Yes

2. Has the statistical analysis been performed appropriately and rigorously?

Reviewer #1: Yes

3. Have the authors made all data underlying the findings in their manuscript fully available?

The PLOS Data policy requires authors to make all data underlying the findings described in their manuscript fully available without restriction, with rare exception (please refer to the Data Availability Statement in the manuscript PDF file). The data should be provided as part of the manuscript or its supporting information, or deposited to a public repository. For example, in addition to summary statistics, the data points behind means, medians and variance measures should be available. If there are restrictions on publicly sharing data—e.g. participant privacy or use of data from a third party—those must be specified.

Reviewer #1: No

**Author comment:** There are ethical and legal restrictions on sharing a de-identified data set. The SUS databases are not open for public review with strict regulations, federal laws and requirements for access. Also, we have sensitive patient information like physical address, location data, specific dates such as, birth dates, death dates and procedures dates. According to the opinion by the Ethics Committee of the University Hospital at the Federal University of São Paulo (HSP/UNIFESP, processes number 2.468.332 and CAAE 81043417.6.0000.5505), the database must be kept confidential. Researchers can contact the authors for more information if wished regarding the analysis. We have now added in a sentence regarding this.

For further information and data requests, follow the contact of the Professor Dr. Mariângela Leal Cherchiglia from the Department of Preventive and Social Medicine, College of Medicine, Federal University of Minas Gerais, Brazil and SUS Collaborating Centre—Technology Assessment & Excellence in Health, College of Pharmacy, Federal University of Minas Gerais, Brazil. E-mail: ccates@ccates.org.br; cherchml@medicina.ufmg.br.

4. Is the manuscript presented in an intelligible fashion and written in standard English?

PLOS ONE does not copyedit accepted manuscripts, so the language in submitted articles must be clear, correct, and unambiguous. Any typographical or grammatical errors should be corrected at revision, so please note any specific errors here.

Reviewer #1: No

**Author comment:** Thank you – we have now corrected all typographical and grammatical errors at revision and hope this is now acceptable.

5. Review Comments to the Author

Please use the space provided to explain your answers to the questions above. You may also include additional comments for the author, including concerns about dual publication, research ethics, or publication ethics. (Please upload your review as an attachment if it exceeds 20,000 characters)

Reviewer #1: The authors have undertaken an impressive amount of work in linking together these databases, and conducting a large number of analyses. I was concerned about potential introduction of immortal time (see major comment 2), for which I’m suggesting some additional analyses (or, if I’ve misunderstood the design, clarifications). The paper also needs a significant amount of editing prior to publishing for readability, and it is very long at the moment. I would consider dropping one or more of the objectives and/or significantly shortening the introduction and discussion. I’m therefore recommending major revisions.

Please note that, based on my expertise, I’ve only been able to review the pharmacoepidemiological aspects of the paper (and not the clinical side).

**Author comments:** Thank you for your help with improving the manuscript. Regarding the potential introduction of immortal time, we have now clarified this and hope this is now acceptable. Also, we accepted your suggestion and we significantly shortened the manuscript.

Major comments

1.The paper contains the results from a large number of objectives (comorbidity development, factors associated with treatment failure, comparative effectiveness, persistence analyses) for a large number of drugs. Have the authors considered splitting the paper into two? Alternatively, I would recommend a significant amount of editing to shorten this paper to a standard length.

**Author comments:** Thank you - we shortened this paper and we focused on factors associated with treatment failure and comparative effectiveness. We hope this is now acceptable.

2.It seems that all patients were required to have at least 6 months of follow-up (row 206-207), however, it then seems patients were followed from the first dispensation. It is unclear to me whether this introduction of immortal time was intentional, and if yes, why the start of follow-up didn’t simply start from 6 months after the first dispensation. I would suggest that the authors evaluate whether this requirement of 6 months records of dispensation had an impact on the results, at least in a sensitivity analysis. Alternatively, if this was a requirement for 6 months of dispensation prior to the start of follow-up (as the first row of the results suggests) then this should be clarified.

**Author comments:** We apologize for the misunderstanding. In fact, it was a requirement for 6 months of dispensation prior to the start of the follow-up. This inclusion criteria were adopted to rule out a wrong diagnosis. As the diagnosis is complex, it was observed (by sample inspection) that patients started treatment as a patient with multiple sclerosis, but within a few months they stopped treatment and changed the diagnosis for another disease. The date of entry in the cohort corresponded to the date of the first dispensation after continuous DMT use during these 6 months. We have clarified this and hope this is now acceptable.

Minor comments

1.Row 257. I’m not sure what the authors refer to when they state they performed a pairwise comparison of logistic regression analysis.

**Author comments:** We apologize for the inconvenience. This statement is not part of the paper. The text has been removed and does not appear in the new version of the manuscript.

2.Table 4. The authors could consider scaling age to present it by 5 or 10 years, so that the effect estimates are easier to interpret.

**Author comments:** Thanks for the suggestion. We have now added this information in Table 4.

3.Table 5. I’m not clear on the adjusted analyses in the propensity score matched cohorts, which is introduced in the results. I assume the additional comorbidities were included as time-updated variables, but this is not described in the methods and should be added.

**Author comments:** Thank you – In fact, the additional comorbidities were included as time-updated variables. We have now added them in the methods and hope this is now acceptable.

4.I would recommend including the number under FU at each time-point per group in each of the KM plots

**Author comments:** Thanks for the suggestion. We have now added this information in all KM plots.

6. PLOS authors have the option to publish the peer review history of their article (what does this mean?). If published, this will include your full peer review and any attached files.

If you choose “no”, your identity will remain anonymous but your review may still be made public.

Do you want your identity to be public for this peer review? For information about this choice, including consent withdrawal, please see our Privacy Policy.

Reviewer #1: No
